# Supplementary material for: Identification of photoperiod-induced specific miRNAs in the adrenal glands of Sunite sheep (Ovis aries)
Source: Front Vet Sci. 2022 Jul 22;9:888207. doi: 10.3389/fvets.2022.888207 (PMC9354845; doi:10.3389/fvets.2022.888207)
Supplement: Supplementary file 1 [file Data_Sheet_1.ZIP › Supplementary file/miRDeep2 file.zip!/miRDeep2 file/Novel_Structure/SP42A3/Novel_669.pdf]

Provisional ID : NC_019481.2_30220
Score total : 1787.9
Score for star read(s) : 3.9
Score for read counts : 1780.4
Score for mfe : 2
Score for randfold : 1.6
Score for cons. seed :
Total read count : 3504
Mature read count : 3497
Loop read count : 0
Star read count : 7

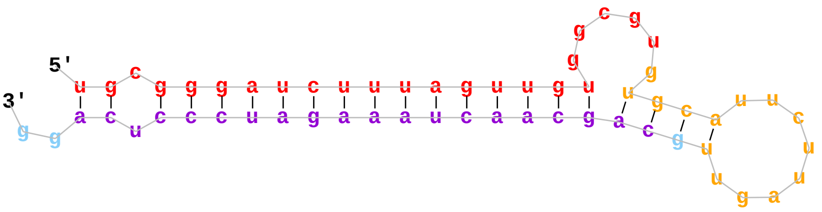

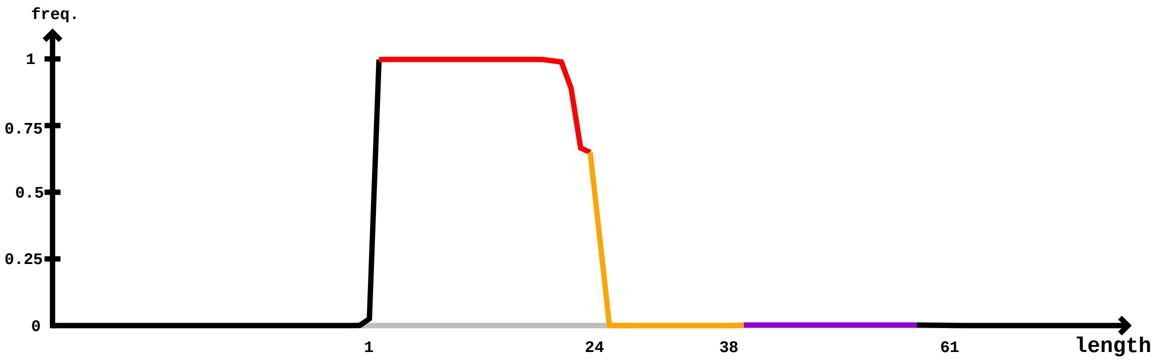

Mature Star

```
5'- aauaacccauaaaagcagccgacguugcggcaugcgggaucuuuaguuguggcgugugcauucuuaguugcagcaacuaaagaucccucaggcugcaaugaagaucagag  -3'   obs
    aauaacccauaaaagcagccgacguugcggcaugcgggaucuuuaguuguggcgugugcauucuuaguugcagcaacuaaagaucccucaggcugcaaugaagaucagag        exp
    ..................(..((((((((.((.((((((((((((((......((((........)))))))))))))))))).)).))))))))..).......   reads   mm   sample
    ..............................caugcgggaucuuuaguuguggc.........................................................   1   0   seq
    ..............................caugcgggaucuuuaguuguggU.........................................................   1   1   seq
    ...............................augcgggaucuuuaguugu............................................................   3   0   seq
    ...............................augcgggaucuuuaguugug...........................................................   8   0   seq
    ...............................augUgggaucuuuaguugug...........................................................   1   1   seq
    ...............................augcgggaucuuuaguugugg..........................................................   10   0   seq
    ...............................augcgggaucuuuaguugugA..........................................................   4   1   seq
    ...............................augcgggaucuuuaguugCgg..........................................................   1   1   seq
    ...............................augcgggaucuuuaguuguggc.........................................................   32   0   seq
    ...............................augcgggaucuuuaguuguAgc.........................................................   1   1   seq
    ...............................augcgggaucuuuaguuguggU.........................................................   18   1   seq
    ...............................augcgggaucuuuaguugugAc.........................................................   1   1   seq
    ...............................augcgggaucuuuaguuguggcg........................................................   5   0   seq
    ...............................augcgggaucuuuaguuguggcC........................................................   2   1   seq
    ...............................auAcgggaucuuuaguuguggcgu.......................................................   1   1   seq
    ................................ugcgggaucuuuaguug.............................................................   1   0   seq
    ................................ugcgggaucuuuaguugC............................................................   8   1   seq
    ................................ugcgggaucuuuaguugA............................................................   5   1   seq
    ................................ugcgggaucuuuaguuguA...........................................................   8   1   seq
    ................................ugcAggaucuuuaguugugg..........................................................   2   1   seq
    ................................ugcgggaucuuuaguugugA..........................................................   79   1   seq
    ................................ugcgggaucuuuaguugugU..........................................................   17   1   seq
    ................................uAcgggaucuuuaguugugg..........................................................   3   1   seq
    ................................ugcgggaucuuuaguugugg..........................................................   226   0   seq
    ................................ugcgggaucuuuaguuguggc.........................................................   710   0   seq
    ................................ugcgggaucuuuaguugCggc.........................................................   3   1   seq
    ................................ugGgggaucuuuaguuguggc.........................................................   1   1   seq
    ................................uAcgggaucuuuaguuguggc.........................................................   3   1   seq
    ................................ugcgggaucuuuaguuguAgc.........................................................   2   1   seq
    ................................ugcgggaucuuuaguugAggc.........................................................   1   1   seq
    ................................ugcgggaucuuuagCuguggc.........................................................   1   1   seq
    ................................ugcgggaucuuuUguuguggc.........................................................   1   1   seq
    ................................ugcAggaucuuuaguuguggc.........................................................   9   1   seq
```

```
                                Mature                             Star
aauaacccauaaaagcagccgacguugcggcaugcgggaucuuuaguuguggcgugugcauucuuaguugcagcaacuaaagaucccucaggcugcaaugaagaucagag
................................ugcgggaucuuuaguugugAc.........................................................   1      1      seq
................................ugcUggaucuuuaguuguggcg........................................................   1      1      seq
................................ugcgAgaucuuuaguuguggcg........................................................   10     1      seq
................................uAcgggaucuuuaguuguggcg........................................................   14     1      seq
................................ugcgggGucuuuaguuguggcg........................................................   19     1      seq
................................ugcgggaucuuuagCuguggcg........................................................   7      1      seq
................................ugcgggaucuuuaguuguggUgu.......................................................   17     1      seq
................................ugcgggaucuuuagCuguggcgu.......................................................   4      1      seq
................................ugcgggaucuuuaguuguggcgu.......................................................   2228   0      seq
................................ugcgAgaucuuuaguuguggcgu.......................................................   9      1      seq
................................ugcgggGucuuuaguuguggcgu.......................................................   6      1      seq
................................uAcgggaucuuuaguuguggcgu.......................................................   9      1      seq
................................ugcgggaucuuuaguuguggcgugug....................................................   1      0      seq
.................................gcgggaucuuuaguugugg..........................................................   1      0      seq
.................................gcUggaucuuuaguuguggcg........................................................   1      1      seq
......................................................................cagcaacuaaagaucccuca....................   7      0      seq
```
